# Ipsilateral and contralateral sensory changes in healthy subjects after experimentally induced concomitant sensitization and hypoesthesia

**DOI:** 10.1186/s12883-017-0839-9

**Published:** 2017-03-23

**Authors:** Elena K. Enax-Krumova, Stephanie Pohl, Andrea Westermann, Christoph Maier

**Affiliations:** 1 Department of Neurology, BG University Hospital Bergmannsheil GmbH, Ruhr University Bochum, Bürkle-de-la-Camp-Platz 1, D-44789 Bochum, Germany; 2 Department of Pain Medicine, BG University Hospital Bergmannsheil GmbH Bochum, Ruhr University Bochum, Bochum, Germany

**Keywords:** Experimental pain model, Capsaicin, Local anesthetics, Neuropathic pain, Quantitative sensory testing, Pain mechanisms, Sensory profiles, Translational pain research, Contralateral sensory changes

## Abstract

**Background:**

In unilateral neuropathic pain. e.g. after peripheral nerve injury, both positive and negative sensory signs occur often, accompanied by minor but equally directed contralateral sensory changes. To mimic this feature, we experimentally aimed to induce concomitant c-fibre sensitization and block in healthy subjects and analyzed the bilateral sensory changes by quantitative sensory testing (QST) using the protocol of the German Research Network on Neuropathic Pain.

**Methods:**

Twenty eight healthy subjects were firstly randomized in 2 groups to receive either topical capsaicin (0.6%, 12 cm^2^, application duration: 15 min.) or a lidocaine/prilocaine patch (25/25 mg, 10 cm^2^, application duration: 60 min.) on the right volar forearm. Secondly, 7–14 days later in the same area either at first capsaicin (for 15 min.) and immediately afterwards local anesthetics (for 60 min.) was applied (Cap/LA), or in inversed order with the same application duration (LA/Cap). Before, after each application and 7–14 days later a QST was performed bilaterally. Statistics: Wilcoxon-test, ANOVA, *p* < 0.05.

**Results:**

Single application of 0,6% capsaicin induced thermal hypoesthesia, cold hypoalgesia, heat hyperalgesia and tactile allodynia. Lidocaine/prilocaine alone induced thermal and tactile hypoesthesia as well as mechanical and cold hypoalgesia, and a heat hyperalgesia (to a smaller extent). Ipsilaterally both co-applications induced a combination of the above mentioned changes. Significant contralateral sensory changes occurred only after the co-application with concomitant sensitization and hypoesthesia and comprised increased cold (Cap/LA, LA/Cap) and mechanical detection as well as cold pain threshold (LA/Cap).

**Conclusion:**

The present experimental model using combined application of capsaicin and LA imitates partly the complex sensory changes observed in patients with unilateral neuropathic pain and might be used as an additional surrogate model. Only the concomitant use both agents in the same area induces both positive and negative sensory signs ipsilaterally as well as parallel contralateral sensory changes (to a lesser extent).

**Trial registration:**

ClinicalTrials.gov Identifier NCT01540877, registered on 23 February 2012.

**Electronic supplementary material:**

The online version of this article (doi:10.1186/s12883-017-0839-9) contains supplementary material, which is available to authorized users.

## Background

Treating neuropathic pain is challenging, especially in the face of the low response rate [1]. One reason for the limited treatment response in neuropathic pain could be that there are different underlying pathomechanisms across all entities [2–6]. One subgroup presents preserved sensory function in combination with thermal hyperalgesia, the other is characterized by predominant sensory loss for thermal and mechanical stimuli. In a third group signs for sensory loss and gain coexist producing combination of hyperalgesia and numbness in the same painful, e. g. in about 70% in postherpetic neuralgia (PHN) or 60% in peripheral nerve injury [7, 8].

Therefore, for translational research appropriate human experimental pain models are needed, mimicking most of the characteristic clinical signs and their above mentioned relevant combination. Yet, most of the human pain models concentrate either on sensory gain (hyperalgesia induced by electrical stimulation, intradermal capsaicin, topical capsaicin or topical menthol) or sensory loss, induced by ischemia or nerve compression [9, 10]. However, none of these surrogate models has documented the concomitant occurrence of sensory loss and gain, although they have been observed both in animal models after unilateral nerve damage and in unilateral pain syndromes (e.g. complex regional pain syndrome (CRPS), trigeminal neuralgia and PHN [11–13]). This contralateral reaction parallel the ipsilateral sensory changes usually in the same direction (i.e. loss and gain, respectively) [14, 15]. Unfortunately, the contralateral change in the human surrogate pain models have not been studied extensively yet. Several potential mechanisms for the mirror-image contralateral changes in patients and in the animal models have been discussed, and the hypothesis, that the these changes are mediated by central nervous mechanisms, e.g. activation of contralateral homonymous neurons or spinal interneurons has been proclaimed as more likely rather than systemic circulating factors, released by the lesioned nerve [14].

The need for experimental designs that mimic clinical signs, including contralateral effects, has been acknowledged as a prerequisite for further research [16]. Also, it has been reported that intracutaneous capsaicin injection in healthy subjects elicited secondary hyperalgesia coexisting with secondary tactile hypoesthesia [17], indicating that the surrogate pain model based on a short lasting intervention is able to induce central nervous plasticity changes in an area outside the topical application, at least ipsilaterally. Therefore, at a first step, we intended to mimic the above mentioned combined gain and loss symptomatology, found in patients with neuropathic pain, in one limited area in healthy subjects. We have examined the sensory alterations after combined C-fiber block and C-fiber sensitization by local anesthetics (LA) and capsaicin 0.6%, respectively, in both different application orders (sensitization of blocked C-fibers and block of sensitized C-fibers) compared to the sensory changes induced by each of both substances alone. Aim of the study was to prove if the concomitant application of capsaicin and LA, inducing C-fiber block and C-fiber sensitization, is superior to the single application of both agents in generating an ipsilateral combination of positive and negative signs, and contralateral sensory changes mirroring the symptoms observed in a subgroup of patients with neuropathic pain.

## Methods

### Study design

**Fig. 1:**
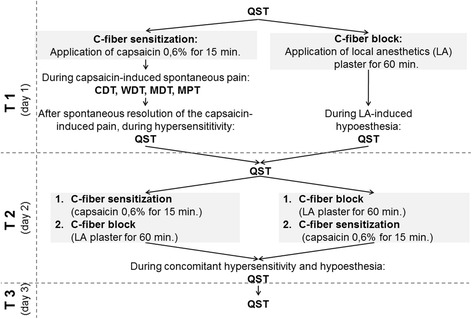
Study design. QST: quantitative sensory testing; CDT, cold detection threshold; MDT, mechanical detection threshold; MPT, mechanical pain threshold; WDT, warm detection threshold

The study consists of two experimental blocks each of them including 2 study arms, performed all together on three sessions with 7–14 days between them (Fig. 1). At first, subjects were randomized to receive either application of topical capsaicin 0.6% or local anesthetics (LA) alone. Quantitative sensory testing (QST) according to the DFNS protocol was performed immediately before and after the LA-application, and 7–14 days later. In the case of capsaicin application, QST was performed additionally immediately before the application, immediately after the application during ongoing capsaicin-induced pain (only thermal and tactile detection, and mechanical pain threshold), as well as after the spontaneous resolution of the capsaicin induced ongoing pain during C-fiber sensitization and 7–14 days later. In the second block after randomization, the subjects received a combined application of either first capsaicin 0.6% and afterwards LA, or both agents in the opposite sequence. QST was performed immediately before the intervention, immediately after that and 7–14 days later. The experiments are described in detail below.

A sample size calculation was performed using unpublished data from a study on the sensory changes after topical application of capsaicin 0.6% using QST according to the DFNS (Binder et al. oral personal communication, Jan/2011) and revealed for a power of 80%, type I error of 0.5 and an estimated drop-out rate of 5% a sample size of at least 14 subjects per study arm.

### Subjects

After approval by the local ethics committee of the Faculty of Medicine, Ruhr-University Bochum (Ref-Nr: 3643-11, 23.02.2011; trial registration number: ClinicalTrials.gov Identifier NCT01540877, registered on 23 February 2012) and written informed consent 30 right-handed healthy volunteers > 18 years old were recruited using a check list established by the IMI-Europain project after medical history appraisal and short clinical examination [18]. Subjects were recruited among students, relatives and hospital staff by SP, who was student herself at the time of recruitment, to avoid coercion. Most of the subjects were recruited by advertisement or by word-of-mouth recommendation by subjects who have already participated in the study. It was explicitly offered to abort the study participation in case of unbearable pain.

Exclusion criteria were defined according to the recently published recommendations by Gierthmühlen et al. [18], i.e. insufficient German language skills, history of any severe internal, neurological or dermatological diseases, substance abuse, manifest psychiatric diseases, chronic and acute pain, any medication intake (except contraceptives in females) regularly or on demand during the last 14 days before study inclusion and during the study period, pregnancy, nursing, abnormal sensory profile in the quantitative sensory testing (QST) with side-to-side differences beyond the normal range [19] at baseline and participation in clinical trials during the last month. Study-specific exclusion criteria were hypersensitivity to lidocaine or other amide-type anesthetics and hereditary or acquired methemoglobinemia.

Two subjects were excluded: one had an abnormal side-to-side differences during the baseline QST, the other developed acute pain due to a metacarpal fracture during the study period. Thus, 28 subjects were enrolled for statistical analysis.

### Experiments

The study design is presented in short in Fig. 1.

#### Day 1

Participants were randomized into two groups after baseline QST. In the first study arm of the first study block (Cap) we unilaterally applied bonded gaze (12 cm^2^) containing 0,4 ml capsaicin solution (0.6% in 45% ethanol, 0.025 M) in the middle of the right volar forearm for 15 min, covered by a transparent plastic dressing to prevent evaporation [20, 21]. The pain intensity was reported on an 11-point numeric rating scale (NRS, 0-10) every 5 min during the application. During acute ongoing pain we assessed the cold, warm and mechanical detection thresholds and the mechanical pain thresholds at the application site and contralateral. After spontaneous resolution of the ongoing capsaicin-induced pain a complete QST was performed in the same areas. The time, when the skin hypersensitivity in the area of capsaicin application recovered, was reported on day 2.

In the second study arm of the first study block a plaster with LA containing 25 mg prilocaine and 25 mg lidocaine (EMLA®, AstraZeneca, 10 cm^2^) was applied in the middle of the right volar forearm for 60 min. Afterwards, a complete QST was performed in the application site and contralateral.

#### Day 2

Seven to fourteen days later after a second randomization and another baseline QST, in the first study arm of the second study block capsaicin was applied for 15 min and then the lidocaine/prilocaine plaster was applied for 60 min (as described above, Cap/LA-group). Ongoing pain intensity was recorded every 5 min during the capsaicin application and every 15 min during the LA application.

In the second study arm of the second study block the lidocaine/prilocaine patch was applied for 60 min and afterwards a capsaicin bonded gaze was applied for another 15 min (as described above, LA/Cap-group). The pain intensity was recorded only during capsaicin application every 5 min.

Immediately after removal of both substances the areas of allodynia and hyperalgesia were mapped (see below) and QST was again performed. The time, when the skin hypersensitivity in the area of capsaicin application recovered, was reported on day 3.

#### Day 3

Another 7–14 days later QST was assessed bilaterally in the previously examined areas.

### Sensory assessment

#### Quantitative sensory testing (QST)

QST was performed according to the standardized protocol of the German Research Network on Neuropathic Pain (DFNS) [19], assessing a complete sensory profile after measuring the skin temperature using an infrared thermometer. The following parameters were assessed exactly following the DFNS protocol: cold and warm detection thresholds (CDT, WDT), cold and heat pain thresholds (CPT, HPT), the ability to discriminate between consecutive warm and cold stimuli (thermal sensory limen, TSL), including any paradoxical heat sensation (PHS, i.e. perceiving cold stimuli as warm), mechanical detection thresholds (MDT), mechanical pain thresholds (MPT), mechanical pain sensitivity (MPS) and temporal summation to repeated pain stimuli (wind-up ratio, WUR), vibration detection thresholds (VDT), pressure pain threshold (PPT) and dynamic mechanical allodynia (DMA). QST was performed at the department’s certified QST lab by a trained investigator (S.P.), according to the published standards for quality assessment [22].

#### Mapping

After removal of all substances, the areas of tactile hypoesthesia and allodynia (using a Q-tip) and pinprick hyperalgesia (using a microfilament, Twin Tip®, 10 g ~ 9,8mN) were assessed, as previously described [23]. The perception areas of tactile hypoesthesia, allodynia and pinprick hyperalgesia were marked on the skin, copied to a transparent sheet and calculated (cm^2^) using AutoCAD (Version 2012).

### Questionnaires

The Hospital Anxiety and Depression Scale (HADS) was assessed in the validated German version, including the degree of depression and anxiousness (abnormal: score > 7) [24, 25]. The Pain Catastrophizing Scale (PCS) was used to determine the relation of negative expectations, emotional distress and pain maintenance (catastrophizers: score > 24, non-catastrophizers: score < 15) [26, 27]. The individual sensitivity to painful events in everyday life situations on the NRS (0-10) was measured by the Pain Sensitivity Questionnaire (PSQ) [28].

### Statistics

QST parameters were transformed into z-values [19] referring to our baseline testing on the volar forearm in all 28 subjects:

$$ \mathrm{z}\hbox{-} \mathrm{value}=\left(\mathrm{mea}{\mathrm{n}}_{\mathrm{single}\ \mathrm{s}\mathrm{ubject}}\hbox{--} \mathrm{mea}{\mathrm{n}}_{\mathrm{baseline}\ \mathrm{healthy}\ \mathrm{s}\mathrm{ubject}\mathrm{s}}\right)/\mathrm{S}{{\mathrm{D}}_{\mathrm{baseline}}}_{\mathrm{healthy}\ \mathrm{s}\mathrm{ubject}\mathrm{s}} $$

A z-score of zero represents the baseline mean. Z-scores > 0 show a higher sensitivity (hyperesthesia, hyperalgesia) compared to the baseline mean, z-values < 0 show a lower sensitivity (hypoesthesia, hypoalgesia). Z-values of 0 ± 1.96 represent the 95% confidence interval of the baseline data.

The data before and after the intervention were compared using Wilcoxon-test. The hyperalgesia and allodynia area sizes and the pain intensities during capsaicin application were compared between the three groups Cap, Cap/LA and LA/Cap, and also between the subgroups of study members showing contralateral sensory alterations and those without any contralateral sensory alterations using ANOVA. *P*-value < 0.05 was considered significant.

## Results

### Dermal reactions

*After application of only capsaicin* all subjects developed ipsilaterally flare and burning ongoing pain, 7 additionally edema and one reported itch.

*After single application of LA* we observed central paleness in the plaster covered skin due to vasoconstriction in 12 of the 14 subjects. Two of them presented flare in the plaster adhesion surface.

*In the Cap/LA-group* all participants developed flare after the capsaicin application, 6 additionally edema and one reported itch. After the LA-application we observed in 5 only flare, in 6 - flare with central paleness and in further 3 - only central paleness.

*In the LA/Cap-group* after the LA-application 4 subjects had no dermal alterations, 2 developed flare and 8 - central paleness. After the capsaicin application all 14 participants developed flare, 6 demonstrated swelling and one reported itch.

### Questionnaires

The PSQ revealed low values, without differences between the study groups (Table 1). The HADS depression score detected normal values. The anxiety score was marginally increased in one subject (8 points) without clinical symptoms. The PCS identified 24 as non-catastrophizer, one was classified as catastrophizer.

**Table 1:** Clinical data

	Part 1. Single substance application		Part 2. Combined substance application^a^	
	Cap-group	LA-group	Cap/LA-group	LA/Cap-group
	(*n* = 14)	(*n* = 14)	(*n* = 14)	(*n* = 14)
gender (female, n (%))	9 (64%)	8 (57%)	9 (64%)	8 (57%)
age (years, mean ± SD)	30 ± 14	30 ± 13	31 ± 13	30 ± 14
BMI (kg/m^2^)	23 ± 2	24 ± 4	23 ± 3	24 ± 3
HADS A-Score	3.9 ± 2.3	2.9 ± 2.0	2.9 ± 2.1	3.8 ± 2.3
HADS D-Score	1.5 ± 1.6	0.6 ± 0.9	0.9 ± 1.2	1.3 ± 1.5
Pain sensitivity score (PSQ), total	2.7 ± 0.9	2.5 ± 0.9	2.5 ± 0.8	2.7 ± 1
Pain catastrophizing scale (PCS), total	7.1 ± 8.2	10.1 ± 7.4	8.5 ± 7.7	8.6 ± 8.2
Duration of acute capsaicin-induced pain (min)	32 ± 7***	not applicable	83 ± 30.8***	48 ± 14***^c^
Mean spontaneous pain intensity after capsaicin gauze removal (NRS 0-10)	6.7 ± 1.3	not applicable	6.7 ± 1.8	6.4 ± 1.9
Duration of reported hypersensitivity after capsaicin application (days)	1 ± 0.5	not applicable	0.8 ± 0.5	0.9 ± 0.5
Area of allodynia (cm^2^)	60 ± 39*	not applicable	32 ± 22*	48 ± 17*
Area of pinprick hyperalgesia (cm^2^)	66 ± 43	not applicable	57 ± 30	54 ± 25
Area of tactile hypoesthesia (cm^2^)	not applicable	9 ± 4	not applicable	not applicable
Skin temperature change after removal of the applied substances (Δ °C)^b^	1.4 ± 1.4	-1.1 ± 1.1**	0.7 ± 1.5	1.8 ± 1.7

*Cap* single capsaicin application, *LA* single local anesthetics application, *Cap/LA* combined application of 1. capsaicin and 2. local anesthetic, *LA/Cap* combined application of 1. local anesthetic and 2. Capsaicin, *HADS* hospital anxiety and depression score, *HADS-A-Score* anxiety score, *HADS-D-Score* depression score, *SD* standard deviation

^a^Participants of the first part of the experiment are also involved in the second part. Therefore, values on the demographic characteristics, HADS, PSQ and PCS are redistributed for the data presentation of the part 2 of the study

^b^Positive values indicate higher skin temperature after the substance application, negative values - lower temperature, respectively

^c^The mean value for the duration of acute capsaicin-induced pain was calculated only based on 5 subjects, the rest 9 subjects were still sensing ongoing pain at the end of the study procedures

*Significant difference (*p* < 0.05) between all three groups (ANOVA)

***Significant difference (*p* < 0.001) between all three groups (ANOVA)

### Intensity of the ongoing pain in the area of capsaicin application

**Fig. 2:**
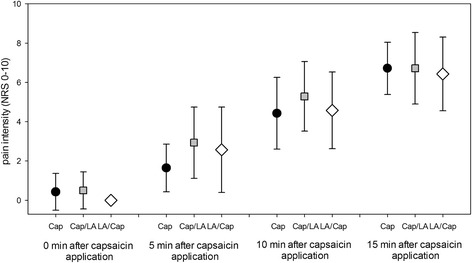
Intensity of ongoing pain during capsaicin application in the group with application of a single agent (*Cap, black*) as well as in the groups with combined application of first capsaicin and then local anesthetics (*Cap/LA, gray*) and of first local anesthetics and then capsaicin (*LA/Cap, white*)

Ongoing pain was reported only ipsilaterally to the capsaicin application. The mean pain intensity in the Cap-group and in both groups with combined application (LA/Cap-group and Cap/LA-group) was similar immediately after topical capsaicin (Table 1, Fig. 2). However, in the LA/Cap-group acute pain terminated in only 5 participants within 48 ± 14 min after the patch removal, while the other 9 subjects perceived ongoing pain until the end of the study procedures. In contrast, in the Cap/LA-group the ongoing pain terminated in all 14 subjects within 83 ± 30.8 min (Chi^2^-Test, *p* < 0.01).

### Ipsilateral area of hyperalgesia, allodynia and hypoesthesia

The pinprick hyperalgesia area was mainly identical to the area of flare, nonetheless, differing from the dynamic allodynia area with high inter-individual variance, but without significant differences between all three groups. The size of the area with allodynia were significantly larger in the LA/Cap-group compared to the Cap/LA-group, but both were significantly smaller than after capsaicin application alone (Table 1). An area of tactile hypoesthesia was found only in the LA-group, mostly identical with the lidocaine/prilocaine plaster size.

### Sensory changes in QST parameter

All data are summarized in Additional file 1: Table S1 and Additional file 2: Table S2.

#### Ipsilateral sensory changes

**Fig. 3:**
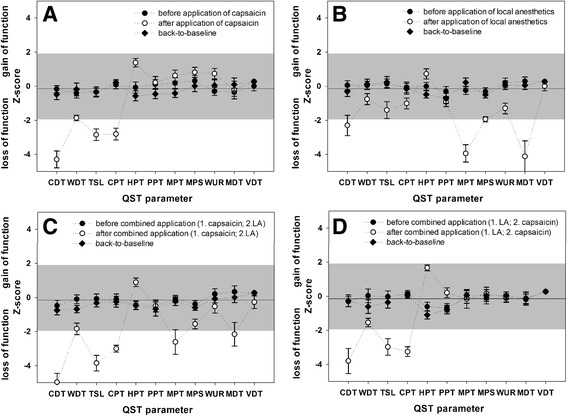
Ipsilateral z-profiles measured before (*black circuits*) and after single application of (**a**) capsaicin or (**b**) local anesthetics, as well as after combined application of (**c**) first capsaicin then local anesthetics and (**d**) first local anesthetics then capsaicin (*white circuits*) as well as back to baseline 7-14 days later (*black diamonds*) on the subjects’ right forearm. Z-values between -1.96 and +1.96 represent the 95% confidential interval of the baseline measurement in the whole group of 28 healthy subjects, z-values greater than 0 demonstrate a sensory gain compared to the group mean of the baseline QST, while z-values less than 0 demonstrate a sensory loss. CDT, cold detection threshold; CPT, cold pain threshold; DMA, dynamic mechanical allodynia; HPT, heat pain threshold; MDT, mechanical detection threshold; MPS, mechanical pain sensitivity; MPT, mechanical pain threshold; NRS, numeric rating scale; PHS, paradoxical heat sensation; PPT, pressure pain threshold; TSL, thermal sensory limen; VDT, vibration detection threshold; WDT, warm detection threshold; WUR, wind-up ratio

*After application of only capsaicin* the sensory profile showed both significant negative signs (for CDT during both acute pain and capsaicin-induced evoked pain after spontaneous pain relief, for WDT and for CPT) as well as positive signs for HPT, MPS and WUR (Fig. 3a). Three subjects reported PHS and 8 showed DMA. *After single LA-application* the sensory profile was dominated by significant sensory loss for several detection and pain thresholds: CDT, MDT, WDT (to a lesser extent) MPT, PPT, MPS and WUR, with the exception of HPT, which significantly decreased (Fig. 3b). *After combined application of both agents* (Cap/LA-group and LA/Cap-group) the thermal detection and pain thresholds were similar to the ones after capsaicin application alone, with additional signs for significant sensory loss for MDT, MPT and MPS (only in the Cap/LA-group) and sensory gain for PPT (only in the LA/Cap-group) (Fig. 3c, d). In both groups of combined application (Cap/LA-group and LA/Cap-group) all subjects developed DMA.

**Fig. 4:**
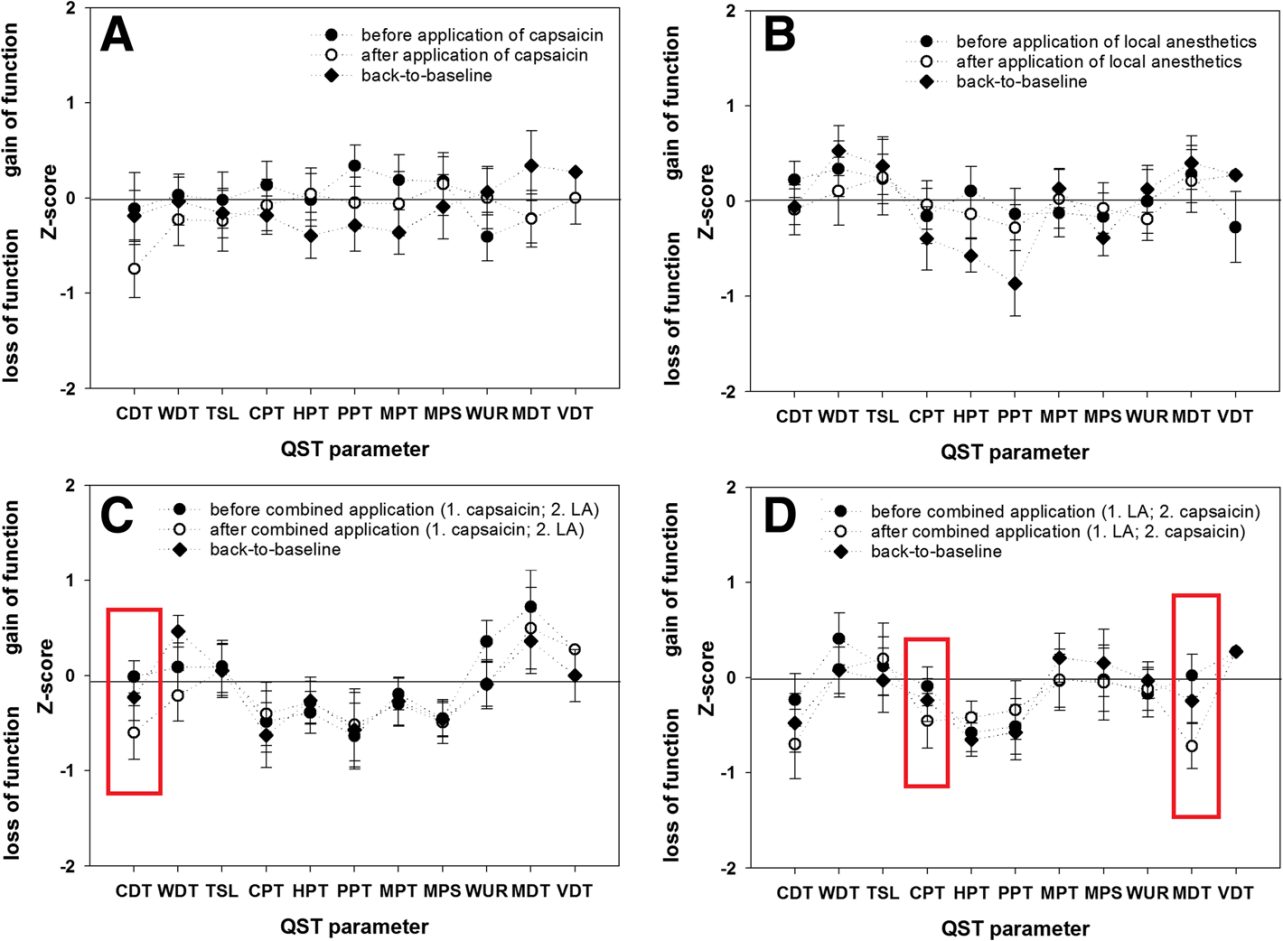
Contralateral z-profiles measured before (*black circuits*) and after single application of (**a**) capsaicin or (**b**) local anesthetics, as well as after combined application of (**c**) first capsaicin then local anesthetics and (**d**) first local anesthetics then capsaicin (*white circuits*) as well as back to baseline 7-14 days later (*black diamonds*) on the subjects’ right forearm. Z-values between -1.96 and +1.96 represent the 95% confidential interval of the baseline measurement in the whole group of 28 healthy subjects, z-values greater than 0 demonstrate a sensory gain compared to the group mean of the baseline QST, while z-values less than 0 demonstrate a sensory loss. *Red borders* indicate significant contralateral changes. CDT, cold detection threshold; CPT, cold pain threshold; DMA, dynamic mechanical allodynia; HPT, heat pain threshold; MDT, mechanical detection threshold; MPS, mechanical pain sensitivity; MPT, mechanical pain threshold; NRS, numeric rating scale; PHS, paradoxical heat sensation; PPT, pressure pain threshold; TSL, thermal sensory limen; VDT, vibration detection threshold; WDT, warm detection threshold; WUR, wind-up ratio

#### Contralateral sensory changes

*After application of only capsaicin* a significant slight increase of only CDT was observed contralateral to the capsaicin application site only during the time of acute ongoing pain (in 10 of 14 subjects), but not during hypersensitivity after the spontaneous recovery of the capsaicin-induced pain (Fig. 4). *After single LA-application* the contralateral detection thresholds remained unchanged. *After combined application of first capsaicin then LA (Cap/LA-group)* CDT again was significantly higher contralaterally (in 11 of 14 participants, Additional file 3: Figure S1A). *After combined application of first LA then capsaicin (LA/Cap-group)* CDT (in 10 of 14 subjects), MDT (in 11 of 14 subjects) and CPT (in 11 of 14 subjects) were significantly higher contralaterally (Additional file 3: Figure S1B). Ipsilateral pain intensity did not correlate with the amount of the contralateral sensory changes of CDT and MDT.

## Discussion

We applied consecutively topical capsaicin and local anaesthetics to induce concomitant sensory gain (e.g. sensitization) and loss (i.e. hypoesthesia). In summary, the ipsilateral application of only capsaicin or only LA induced the expected ipsilateral sensory changes. Capsaicin application had only a short-lasting effect on the CDT on the contralateral side, which increased only during the short period, in which capsaicin led to ongoing pain. In contrast, the application of LA had no effect on the contralateral sensory function. Interestingly, after combined application of capsaicin and LA, independently of the sequence, CDT increased contralaterally for a longer period than the period with the ongoing pain. Additionally, in the LA/Cap-group the MDT increased and CPT decreased contralaterally to the application site. All contralateral changes followed the same direction as the changes observed in the corresponding thresholds on the application site but to a lesser extent. Positive ipsilateral signs were not mirrored.

### The challenge of translational pain research

Hitherto reported human experimental pain models have focused on mechanisms inducing sensory gain or loss ipsilateral. Examples of a surrogate model are the topical or intradermal capsaicin application, evoking plus signs like pinprick hyperalgesia and allodynia [7] and topical menthol in high concentration inducing cold hyperalgesia, but both mostly no hypoesthesia (except for cold hypoesthesia after capsaicin application) [29, 30]. On the other hand, the transient ischemic nerve fiber block induced only sensory loss [31–33]. To our knowledge, there was no such a human pain model focusing on the induction of concomitant sensory loss and gain signs, which were described on the capsaicin model and the electrical stimulation [17, 34].

Indeed, the phenomenon of concomitant sensory loss and gain is frequent in patients with chronic neuropathic pain and has recently been described in about 50% of different neuropathic pain in different entities [7, 8], particularly in PHN [35] and after peripheral nerve injury, but also in CRPS [11, 36, 37] and central post-stroke pain [38]. Thus, our experimental pain model with transient sensory loss and gain mimics a part of the sensory dysfunction of neuropathic pain states. The topical substance application makes it easy to handle. Though, this pain model, as all others previously described, can imitate only some aspects of the clinical states but not to the complex symptomatology of chronic pain [10], because it is obvious that for ethical reasons a lesion or disease of the somatosensory system in healthy volunteers cannot be induced. This may generally limit the use of human surrogate models for neuropathic pain and represents one critical point for any translational pain research. For instance, they are usually based on short acting interventions, which are hard to compare with features of neuropathic pain that has developed chronically due to lesion of the somatosensory system, leading to well established mechanisms such as central and peripheral sensitization, enhanced peripheral fiber excitability, abnormal alteration in cord circuitry, etc.

### Ipsilateral sensory changes

We used topical capsaicin as an established model to induce positive sensory signs in healthy subjects. The ipsilateral heat hyperalgesia, enhanced MPS and DMA after application of only capsaicin develop due to peripheral sensitization after TRPV1-receptor activation in epidermal C-fibers [38–40]. The secondary hyperalgesia and allodynia around the application site suggest central sensitization, even after the short lasting application [32]. The previously described tactile hypoesthesia in the secondary area after capsaicin application indicates additionally selective spinal inhibition of mechanoreceptive nerve fibres following the selective excitation of capsaicin-sensitive C-fibre nociceptors [17]. Interestingly, capsaicin induced not only positive, but also negative thermal signs. Remarkably, the largest change from baseline after capsaicin application was the increase of the cold detection threshold, although capsaicin has primary effect on TRPV1, and as such is expected to affect warm and heat perception most significantly. Our results of cold hypoesthesia are in line with a previous study [41]; however, the underlying mechanisms are unclear. Several peripheral mechanisms for the inhibition of cold sensation by capsaicin have been hypothesized [41]. A coexpression of TRPV1 and TRPM8 receptors has been suggested, where the activation of one of the receptors probably leads to opposed functional changes of the other [41–43]. On the other hand, the capsaicin-induced inflammation leads to release of bradykinin and prostaglandin E and subsequent shift of the threshold temperature of TRPM8 expressing neurons to colder values mediated by protein kinase A [41, 44].

On the other hand, in order to induce negative sensory signs we applied LA. The ipsilateral thermal and tactile hypoesthesia and pinprick hypoalgesia after LA-application alone result from blocking sodium channels on C-fibres [45, 46]. Yet, we observed a HPT decrease also after lidocaine/prilocaine application. This finding is in contrast to our published data on sensory changes after pure lidocaine (5%) application [46], but in line with a previous study, in which lidocaine sensitized normal skin for heat [47]. The heat hyperalgesia seems to underlie a lidocaine-induced activation and sensitization of TRPV1 and TRPA1 [48].

Applying both substances, we could block sensitized c-fibers and vice versa sensitize blocked c-fibers in healthy subjects, depending on the application sequence. The ongoing pain intensity after capsaicin 0.6% was not influenced by the LA-application immediately before capsaicin, as previously described [49]. However, the LA-application after capsaicin accelerated the recovery of capsaicin-induced ongoing pain. In the Cap/LA-group the capsaicin-induced mechanical hyperalgesia was reversible after LA-application, inducing mechanical hypoesthesia and hypoalgesia. Interestingly, in the LA/Cap-group the LA-induced mechanical hypoesthesia and hypoalgesia rose to normal values after capsaicin application and the mean allodynia area was significantly larger than in the Cap/LA-group. We have previously demonstrated that LA in form of a lidocaine (5%) patch induced only a partial small fiber block of unpredictable extent [46]. The different kinetics of the LA-induced sensory changes in the present study after co-application of LA and capsaicin suggests that the sensitization after TRPV1-activation was able to overact the LA-induced block.

### Contralateral sensory changes

A slightly increased CDT, MDT and CPT were observed also in the contralateral mirror skin area (to a lesser extent compared to ipsilateral) which might suggest central involvement in the stimulus processing. The contralateral sensory changes observed in our model indicate again similarity to the clinical observations, likewise in CRPS [11], unilateral osteoarthritis pain [50] and unilateral PHN [12]. Experimental animal studies have already described congruent contralateral reactions after unilateral nerve lesion [14]. Contralateral sensory changes have been also reported in humans, however assessing only single modalities. Bilateral pinprick hyperalgesia and tactile allodynia following unilateral capsaicin injection was measured in healthy subjects and patients with rheumatoid arthritis [51]. Cold hypoesthesia and hypoalgesia as well as tactile hypoesthesia after unilateral topical capsaicin application in healthy subjects have also been described [41, 52].

Exact determination of the underlying mechanisms for the development of the contralateral sensory changes is currently impossible. Persistent pain is supposed to suppress the primary somatosensory cortical activity that normally responds to innocuous tactile sensations [52]. The significantly increased CDT after application of only capsaicin in our study only during ongoing pain supports this hypothesis. However, we cannot exclude that psychological aspects play a role in the development of the contralateral sensory changes, e.g. some effect of distraction during ongoing pain ipsilaterally to the intervention cannot be excluded. On the other hand, the ongoing pain intensity after capsaicin alone and the extent of contralateral changes were not related. Further on, one might argue that the contralateral findings could be explained by a simple ‘anticipatory’ response, where the subject tends to respond on the contralateral side similarly as on the treated side. However, this was the case only for the detection thresholds in the case of combined application of local anesthetics and capsaicin ipsilaterally, whereas the much more pronounced positive signs after capsaicin application and also the ipsilateral hypoesthesia after lidocaine application were not mirrored. Thus, this hypothesis seems not to be probable. A further potential mechanism for the contralateral sensory loss may be a supraspinal descending inhibition of neuronal dorsal horn activity responses following nociceptive input [53]. The contralateral hypoesthesia may also be facilitated due to a contrast phenomenon compared to the intensely perceived painful stimulus ipsilateral. Hitherto, this concept was proposed for the primary visual perception [54], but may also play a role in nociceptive processing as thalamic filtering mechanism [55, 56]. However, the pain intensity and the extent of contralateral changes after combined capsaicin and LA-application were also unrelated, while the hyperalgesia and allodynia areas were even smaller than after application of only capsaicin. Cortical alterations may also be induced by maladaptive stimulus processing affecting central plasticity [23]. Furthermore, commissural interneurons on spinal level may transmit sensory changes between both body sides [14].

Although the changes we have observed in the present study were statistically significant in the group analysis, they were absent in some subjects and are likely to be of different magnitude compared to the clinical cases of neuropathic pain. The less pronounced contralateral sensory changes in our model compared to results in patients with neuropathic pain may be explained by the difference between changes induced by acute and moderate pain in healthy subjects (as in our model) and by chronic neuropathic pain. Additionally, the exact temporal maintenance of the sensory alterations and the exact anatomical spread of the contralateral changes in our study are unknown (except for the fact, that they are reversible after 7-14 days). We did not include a study arm with placebo intervention and neither the investigator nor the subjects were blinded, and this might be a possible limitation based on the observation bias. However, we refrained from that, because a blinding could not be possible due to the pronounced spontaneous sensory changes ipsilaterally following the application of local anesthetics and/or capsaicin, which were obvious for all subjects. An important inclusion criterion was that all subjects were naïve for QST and were not informed about the study hypotheses, especially the focus on contralateral changes, thus they were not biased by certain expectations of the subjects. The above potential mentioned observation bias is expected to be of only minor extend as all assessed parameters of the QST are based on the subjects’ response.

## Conclusion

In summary, the presented human model with combined application of capsaicin and LA might be an additional surrogate model for unilateral neuropathic pain, because it mimics partly its complex symptomatology. Only the concomitant use both agents in the same area induces both positive and negative sensory signs ipsilaterally as well as parallel contralateral sensory changes. Thus, this model may be useful to analyse the anti-hyperalgesic mechanisms of pharmacological and non-medical intervention in the difficult treatment of neuropathic pain.

## Additional files

Additional file 1: Table S1. A. QST data after single substance application (site of application; data are presented as median (range)). **B.** QST data after combined substance application (site of application; data are presented as median (range)). (DOCX 35 kb)

Additional file 2: Table S2. A. QST data after single substance application (contralateral to the application site; data are presented as median (range)). **B.** QST data after combined substance application (contralateral to the application site; data are presented as median (range)). (DOCX 34 kb)

Additional file 3: Figure S1. (**A**) Contralateral changes in the cold detection threshold of all subjects before and after application of a combination of capsaicin and local anesthetics as well as 7-14 days later. (**B**) Contralateral changes in the mechanical detection threshold of all subjects before and after application of capsaicin and local anesthetics as well as 7–14 days later. Gray circuits show the calculated group means. Red borders indicate significant changes. (TIF 1022 kb)

## Abbreviations

CDT: Cold detection threshold
CPT: Cold pain threshold
CRPS: Complex regional pain syndrome
DFNS: German Research Network on Neuropathic Pain (Deutscher Forschungsverbund Neuropathischer Schmerz)
DMA: Dynamic mechanical allodynia
HPT: Heat pain threshold
MDT: Mechanical detection threshold
MPS: Mechanical pain sensitivity
MPT: Mechanical pain threshold
PHN: Postherpetic neuralgia
PHS: Paradoxical heat sensation
PPT: Pressure pain threshold
QST: Quantitative sensory testing
TSL: Thermal sensory limen
VDT: Vibration detection threshold
WDT: Warm detection threshold
WUR: Wind-up ratio
