# Supplementary material for: Ipsilateral and contralateral sensory changes in healthy subjects after experimentally induced concomitant sensitization and hypoesthesia
Source: BMC Neurol. 2017 Mar 23;17:60. doi: 10.1186/s12883-017-0839-9 (PMC5364678; doi:10.1186/s12883-017-0839-9)
Supplement: Supplementary file 1 — QST data after single substance application (site of application; data are presented as median (range)). B. QST data after combined substance application (site of application; data are presented as median (range)). (DOCX 35 kb) [file 12883_2017_839_MOESM1_ESM.docx]

Additional file 1: Table S1.

**A.** QST data after single substance application (site of application; presented as median (minimum…maximum))

|  | Cap-group | | | | LA-group | | |
| --- | --- | --- | --- | --- | --- | --- | --- |
|  | Baseline 1 | After capsaicin application during acute pain | After acute pain relief during hypersensitivity | After 7 to 14 days | Baseline 1 | After local anesthetics | After 7 to 14 days |
| CDT (°C from baseline) | -0,92  (-1,83...-0,47) | **-2,38**  **(-27,57...-0,5)^**^** | **-6,33**  **(-32...-1,37)**^***^ | -0,97  (-3,03...-0,53) | -0,83  (-1,87...-0,4) | **-2,03**  **(-10,4...-0,5) ^**^** | -0,82 (-2,5...-0,53) |
| WDT (°C from baseline) | 1,75  (1,1...5,4) | 2,7  (1,3...4,1) | **4,53**  **(2...5,17)** ^**^ | 1,73  (0,63...3,9) | 1,72  (0,97...3,83) | **2,18 (1,07...6,63)^*^** | 1,67 (0,6...3,9) |
| TSL (°C) | 3,42  (1,73...7,83) | not assessed | **11,18 (4,87...40,47)** ^***^ | 3,55  (1,77...7,13) | 2,78  (1,53...6,33) | **4,73 (1,37...22,57) ^**^** | 2,45 (1,33...7,13) |
| PHS (n) | 0 (0...0) | not assessed | 0 (0...3) | 0 (0...0) | 0 (0...0) | 0 (0...0) | 0 (0...0) |
| CPT (°C) | 25,17  (10...28,8) | not assessed | **2,65 (0...24,3)^***^** | 25,58 (10,9...29,7) | 25,75 (5,17...28,87) | **19,03 (4,9...26,17) ^**^** | 24,25 (2,9...29,7) |
| HPT (°C) | 42,2 (35,33...46,77) | not assessed | **36,6 (33,13...43,4)^**^** | 44,13  (37,9...46) | 41,53 (37,23...44,83) | **39,05 (34,43...43,5)^*^** | 42,8 (38,6...46) |
| MDT (mN) | 1,2  (0,5...3,03) | 1,15 (0,18...3,25) | 1,41  (0,22...6,5) | 0,93  (0,18...2) | 0,71  (0,47...2,14) | **5,33 (0,54...194,01) ^***^** | 1,19 (0,29...2) |
| MPT (mN) | 32  (11,31...90,51) | 38,08 (11,31...84,45) | 26,92 (7,46...78,79) | 42,22 (27,86...128) | 45,25 (22,63...119,43) | **450,01 (24,25...724,08) ^***^** | 32 (16...128) |
| MPS (NRS 0-100) | 0,61  (0,28...6,42) | not assessed | **1,15**  **(0,26...7,19)^*^** | 0,34  (0,14...1,51) | 0,43  (0,14...2,37) | **0,04**  **(0...0,27) ^***^** | 0,39 (0,12...1,51) |
| DMA | 0 (0...0) | not assessed | **0,26 (0...5,49) ^**^** | 0 (0...0) | 0 (0...0) | 0 (0...0,17) | 0 (0...0) |
| WUR (ratio) | 2,12  (1,21...3,8) | not assessed | **3,06 (1,67...8,53)^***^** | 2,28  (1,13...5,6) | 2,36  (1,22...5) | **1,5**  **(1...2,22)^*^** | 2,2 (1,6...5,6) |
| VDT (n/8) | 8 (7,67...8) | not assessed | 8 (8...8) | 8 (8...8) | 8 (8...8) | 8 (7,67...8) | 8 (8...8) |
| PPT (kPa) | 325,37 (225,63...640,92) | not assessed | 346,62 (215,82...703,05) | 447,99 (281,22...719,4) | 389,13 (268,14...591,87) | **431,64 (304,11...768,45) ^*^** | 418,56 (284,49...719,4) |

**B.** QST data after combined substance application (site of application; presented as median (minimum…maximum))

|  | Cap/LA-group (1.capsaicin and 2.local anesthetics) | | | LA/Cap-group (1.local anesthetics and 2. capsaicin) | | |
| --- | --- | --- | --- | --- | --- | --- |
|  | Baseline 2 | After combined application | After 7 to 14 days | Baseline 2 | After combined application | After 7 to 14 days |
| CDT (°C from baseline) | -0,92  (-3,03...-0,53) | **-8,83**  **(-32...-1,77)^###^** | -1,15  (-4,47...-0,7) | -0,87  (-2,83...-0,53) | **-6,68**  **(-32...-0,93) ^###^** | -0,83  (-3,53...-0,5) |
| WDT (°C from baseline) | 1,78  (0,6...3,53) | **4**  **(0,8...7,9) ^###^** | 2,1  (1,23...4,43) | 1,52  (0,63...10,9) | **3,83**  **(1,77...5,8) ^#^** | 2,05 (0,83...14,9) |
| TSL (°C) | 3,4  (1,63...10) | **17,97 (2,53...45,43) ^###^** | 3,58  (1,5...9,23) | 2,98  (1,33...10,3) | **13,28 (3,67...42,5) ^###^** | 3,52 (1,53...20,1) |
| PHS (n) | 0 (0...0) | 0 (0...3) | 0 (0...0) | 0 (0...0) | 0 (0...1) | 0 (0...0) |
| CPT (°C) | 25,18 (2,9...28,43) | **3,25**  **(0...14,33) ^###^** | 24,88 (3,77...27,63) | 25,17 (10,9...29,7) | **0**  **(0...24,37) ^###^** | 25,67 (15,13...29,3) |
| HPT (°C) | 43,33 (37,9...46) | **37,23 (35,5...42,77) ^##^** | 42,8 (38,27...47,67) | 44,12 (38,6...47,53) | **36,12 (33,9...39,9) ^###^** | 44,72 (39,73...49,57) |
| MDT (mN) | 0,97 (0,18...2,83) | **3,38**  **(0,27...64) ^##^** | 1,32 (0,35...2,14) | 1,23  (0,29...4) | 1,34  (0,22...3,25) | 1,28 (0,29...3,03) |
| MPT (mN) | 39,49 (19,7...128) | **247,33 (25,99...724,08) ^##^** | 43,74 (19,7...119,43) | 36,85 (16...137,19) | 38,08 (6,96...222,86) | 39,4 (14,93...137,19) |
| MPS (NRS 0-100) | 0,35 (0,13...1,51) | **0,07**  **(0...1,15) ^##^** | 0,31 (0,14...1,14) | 0,39  (0,12...5,41) | 0,85  (0,11...4,34) | 0,53 (0,13...4,89) |
| DMA | 0 (0...0) | **0,04 (0...1,32) ^#^** | 0 (0...0) | 0 (0...0) | **0,42 (0...6,71) ^###^** | 0 (0...0,02) |
| WUR (ratio) | 2,41  (1,2...5,6) | 2  (1...3,4) | 2,47  (1,13...4) | 2,2  (1,13...5,22) | 2,23  (1,2...4,64) | 2,26  (1,29...4) |
| VDT (n/8) | 8 (8...8) | 8 (7,67...8) | 8 (8...8) | 8 (8...8) | 8 (8...8) | 8 (8...8) |
| PPT (kPa) | 367,88 (281,22...719,4) | 380,96 (248,52...804,42) | 405,48 (251,79...817,5) | 449,63 (284,49...578,79) | **315,56 (255,06...618,03) ^##^** | 449,63 (277,95...621,3) |

CDT: cold detection threshold; WDT: warm detection threshold; TSL: thermal sensory limen; PHS: paradoxical heat sensation; CPT: cold pain threshold; HPT: heat pain threshold; MDT: mechanical detection threshold; MPT: mechanical pain threshold; MPS: mechanical pain sensitivity; DMA: dynamic mechanical allodynia; WUR: wind-up ratio; VDT: vibration detection threshold; PPT: pressure pain threshold

^*^ Significant difference (p<0.05) compared to baseline 1 (WilcoxonTest).

^**^ Significant difference (p<0.01) compared to baseline 1 (WilcoxonTest).

^***^ Significant difference (p<0.001) compared to baseline 1 (WilcoxonTest).

^#^ Significant difference (p<0.05) compared to baseline 2 (WilcoxonTest).

^##^ Significant difference (p<0.01) compared to baseline 2 (WilcoxonTest).

^###^ Significant difference (p<0.001) compared to baseline 2 (WilcoxonTest).
