# Supplementary material for: Ipsilateral and contralateral sensory changes in healthy subjects after experimentally induced concomitant sensitization and hypoesthesia
Source: BMC Neurol. 2017 Mar 23;17:60. doi: 10.1186/s12883-017-0839-9 (PMC5364678; doi:10.1186/s12883-017-0839-9)
Supplement: Supplementary file 2 — QST data after single substance application (contralateral to the application site; data are presented as median (range)). B. QST data after combined substance application (contralateral to the application site; data are presented as median (range)). (DOCX 34 kb) [file 12883_2017_839_MOESM2_ESM.docx]

Additional file 2: Table S2.

**A.** QST data after single substance application (contralateral to the application site; presented as median (minimum…maximum))

|  | Cap-group | | | | LA-group | | |
| --- | --- | --- | --- | --- | --- | --- | --- |
|  | Baseline 1 | After capsaicin application during acute pain | After acute pain relief during hypersensitivity | After 7 to 14 days | Baseline 1 | After local anesthetics | After 7 to 14 days |
| CDT (°C from baseline) | -0,8  (-5,93...-0,43) | **-1,45**  **(-3,53...-0,63)*** | -1,25  (-3,9...-0,53) | -0,8  (-4...-0,53) | -0,77  (-1,5...-0,4) | -0,77  (-2,5...-0,5) | -0,82  (-1,53...-0,53) |
| WDT (°C from baseline) | 1,58 (1,17...3,87) | 2,27 (0,67...6,57) | 2,07  (0,7...4,4) | 1,75 (0,9...4,5) | 1,42  (0,67...4,3) | 1,75 (0,53...6,27) | 1,23  (0,73...2,9) |
| TSL (°C) | 2,92 (1,67...11,17) | not assessed | 3,48  (1,1...9,37) | 2,98 (1,9...8,6) | 2,95  (1...6,03) | 2,87 (0,7...9,67) | 2,65 (0,97...5,97) |
| PHS (n) | 0 (0...0) | not assessed | 0 (0...0) | 0 (0...0) | 0 (0...0) | 0 (0...0) | 0 (0...0) |
| CPT (°C) | 26,15 (7,03...28,47) | not assessed | 24,72 (8,07...27,97) | 22,37 (13,43...28,07) | 24,5 (6,23...28,43) | 25,42 (9,7...29,17) | 23,03 (2,7...29,9) |
| HPT (°C) | 42 (35,8...46,77) | not assessed | 42  (36,1...45,3) | 42,75 (37,73...46,8) | 41,48 (36,47...45,27) | 41,97 (35,73...45,1) | 43,07 (39,77...46,6) |
| MDT (mN) | 1,28 (0,32...3,25) | 1,37 (0,38...4,29) | 1,37 (0,44...3,25) | 0,98 (0,2...4,29) | 0,97 (0,19...2,46) | 1,07 (0,25...2,3) | 0,81  (0,33...2,3) |
| MPT (mN) | 34,63 (12,13...97,01) | 35,7 (5,66...157,59) | 39,4 (13,93...256) | 46,88 (16...111,43) | 39,49 (24,25...147,03) | 34,38 (18,38...103,97) | 37,11 (22,63...103,97) |
| MPS (NRS 0-100) | 0,53 (0,23...3,91) | not assessed | 0,41  (0,2...6,61) | 0,38 (0,14...6,24) | 0,45 (0,14...2,82) | 0,42 (0,16...3,78) | 0,44 (0,13...1,25) |
| DMA | 0 (0...0,04) | not assessed | 0 (0...0) | 0 (0...0) | 0 (0...0) | 0 (0...0) | 0 (0...0,03) |
| WUR (ratio) | 1,92 (1,11...3,89) | not assessed | 2,3  (1,35...7) | 2,4  (1,29...4) | 2,06 (1,23...5,33) | 2,14 (1,27...3,4) | 2,2  (1,6...4,4) |
| VDT (n/8) | 8 (7,67...8) | not assessed | 8 (7,67...8) | 8 (8...8) | 8 (7,67...8) | 8 (8...8) | 8 (8...8) |
| PPT (kPa) | 323,73 (248,52...555,9) | not assessed | 346,62 (268,14...657,27) | 366,24 (281,22...654) | 374,42 (264,87...529,74) | 382,59 (274,68...588,6) | 461,07 (268,14...735,75) |

**B.** QST data after combined substance application (contralateral to the application site; presented as median (minimum…maximum))

|  | Cap/LA-group (1.capsaicin and 2.local anesthetics) | | | LA/Cap-group (1.local anesthetics and 2. capsaicin) | | |
| --- | --- | --- | --- | --- | --- | --- |
|  | Baseline 2 | After combined application | After 7 to 14 days | Baseline 2 | After combined application | After 7 to 14 days |
| CDT (°C from baseline) | -0,77  (-1,53...-0,53) | **-1,17**  **(-2,67...-0,43)^#^** | -1,08  (-1,7...-0,5) | -0,82  (-4...-0,53) | **-1,03**  **(-4,9...-0,5)^#^** | -0,92  (-3,87...-0,53) |
| WDT (°C from baseline) | 1,53  (0,73...2,97) | 1,78  (0,83...4,4) | 1,48 (0,83...2,27) | 1,53 (0,73...2,97) | 1,82  (0,73...4,17) | 1,67 (0,9...3,33) |
| TSL (°C) | 2,9  (0,97...6,5) | 3,02  (1,17...6,27) | 2,98 (1,23...7,13) | 2,93 (1,47...8,6) | 2,77  (0,93...8,83) | 3,12 (1,27...10,53) |
| PHS (n) | 0 (0...0) | 0 (0...0) | 0 (0...0) | 0 (0...0) | 0 (0...0) | 0 (0...0) |
| CPT (°C) | 21,17 (2,7...28,07) | 24,22  (3,9...27,5) | 21,8 (1...26,67) | 23,23 (13,43...29,9) | **23,27 (4,67...27,57)^#^** | 23,18 (10,93...29) |
| HPT (°C) | 42,52 (37,73...46,6) | 42,88 (36,97...44,8) | 42,58 (36,4...46,73) | 43,38 (38,23...46,8) | 42,55 (38,17...46,27) | 43,37 (39,33...46,4) |
| MDT (mN) | 0,71  (0,2...4,29) | 0,82  (0,18...4) | 1,11 (0,23...1,74) | 1,11 (0,5...2,3) | **1,59 (0,62...3,03)^#^** | 1,28 (0,54...3,03) |
| MPT (mN) | 43,74 (25,99...103,97) | 47,1  (19,7...128) | 48,5 (19,7...111,43) | 45,25 (16...111,43) | 35,53 (17,15...181,02) | 35,53 (12,13...97,01) |
| MPS (NRS 0-100) | 0,44  (0,13...1,25) | 0,39  (0,04...1,57) | 0,35 (0,14...1,32) | 0,38 (0,14...6,24) | 0,41  (0,06...6,51) | 0,54 (0,14...7,08) |
| DMA | 0 (0...0) | 0 (0...0,03) | 0 (0...0,06) | 0 (0...0,03) | 0 (0...0) | 0 (0...0) |
| WUR (ratio) | 2,52  (1,74...4) | 2,08  (1,33...6) | 2,3  (1,33...4,8) | 1,94 (1,29...4,4) | 2,16  (1,27...3,64) | 2,14 (1,57...3,89) |
| VDT (n/8) | 8 (8...8) | 8 (8...8) | 8 (7,67...8) | 8 (8...8) | 8 (8...8) | 8 (8...8) |
| PPT (kPa) | 384,23 (281,22...735,75) | 356,43 (268,14...768,45) | 404 (252...870) | 421,83 (268,14...654) | 389,13 (251,79...598,41) | 433 (262...608) |

CDT: cold detection threshold; WDT: warm detection threshold; TSL: thermal sensory limen; PHS: paradoxical heat sensation; CPT: cold pain threshold; HPT: heat pain threshold; MDT: mechanical detection threshold; MPT: mechanical pain threshold; MPS: mechanical pain sensitivity; DMA: dynamic mechanical allodynia; WUR: wind-up ratio; VDT: vibration detection threshold; PPT: pressure pain threshold

^*^ Significant difference (p<0.05) compared to baseline 1 (Wilcoxon Test).

^#^ Significant difference (p<0.05) compared to baseline 2 (Wilcoxon Test).
