# Supplementary figures and images for: Ipsilateral and contralateral sensory changes in healthy subjects after experimentally induced concomitant sensitization and hypoesthesia
Source: BMC Neurol. 2017 Mar 23;17:60. doi: 10.1186/s12883-017-0839-9 (PMC5364678; doi:10.1186/s12883-017-0839-9)

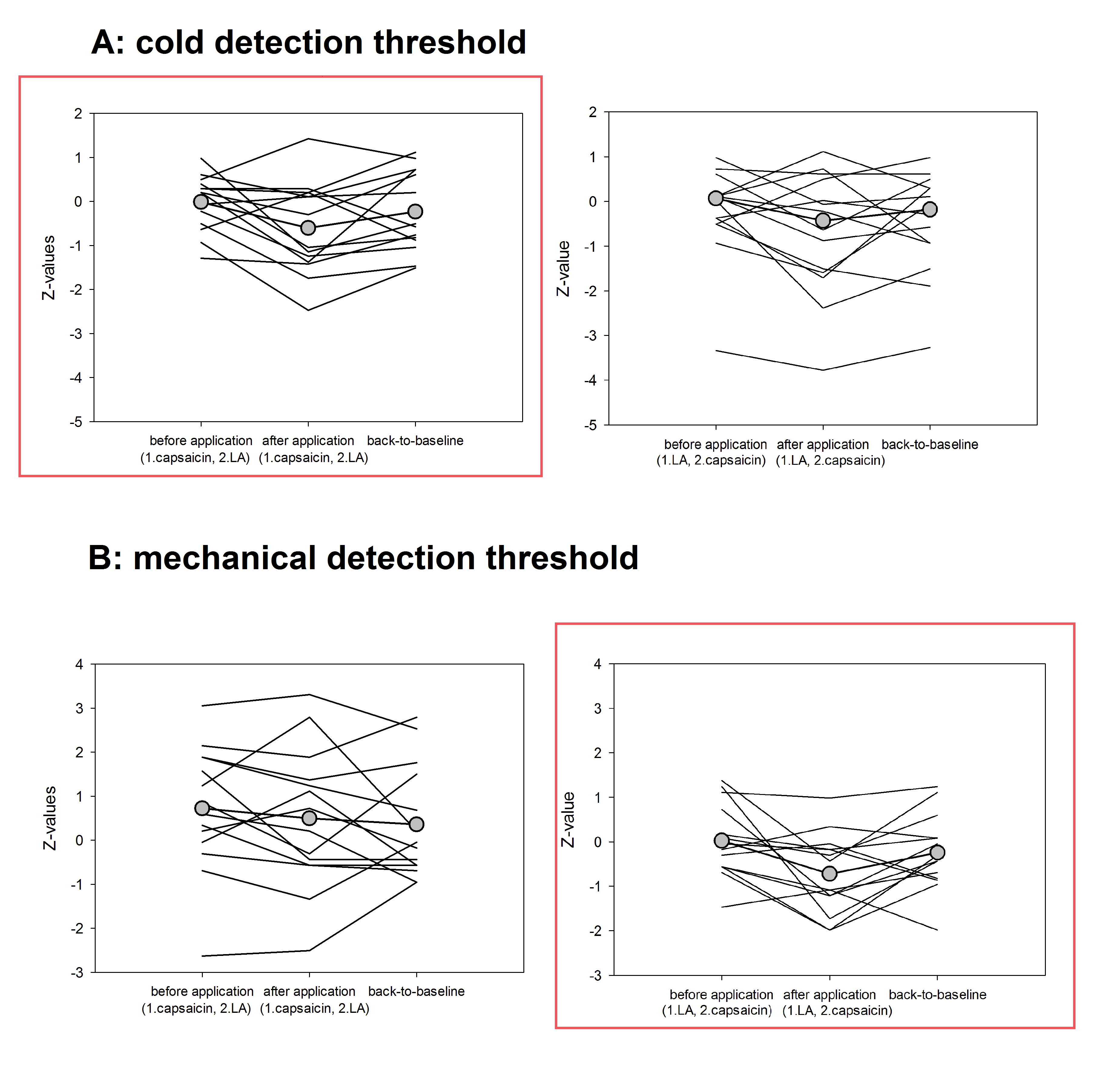

Supplement: Supplementary file 3 — (A) Contralateral changes in the cold detection threshold of all subjects before and after application of a combination of capsaicin and local anesthetics as well as 7-14 days later. (B) Contralateral changes in the mechanical detection threshold of all subjects before and after application of capsaicin and local anesthetics as well as 7–14 days later. Gray circuits show the calculated group means. Red borders indicate significant changes. (TIF 1022 kb) [file 12883_2017_839_MOESM3_ESM.tif]
